# Pathological Investigations of Intracranial Atherosclerosis Using Multiple Hypercholesterolemic Rabbit Models

**DOI:** 10.3389/fendo.2022.834207

**Published:** 2022-05-27

**Authors:** Xiangming Tang, Manabu Niimi, Huanjin Zhou, Lu Chen, Yajie Chen, Haizhao Yan, Masashi Shiomi, Jianglin Fan

**Affiliations:** ^1^ Department of Molecular Pathology, Faculty of Medicine, Interdisciplinary Graduate School of Medicine, University of Yamanashi, Yamanashi, Japan; ^2^ School of Biotechnology and Health Sciences, Wuyi University, Jiangmen, China; ^3^ Key Laboratory of Regenerative Biology, South China Institute for Stem Cell, Biology and Regenerative Medicine, Guangzhou Institutes of Biomedicine and Health, Chinese Academy of Sciences, Guangzhou, China; ^4^ Institute for Experimental Animals, Kobe University School of Medicine, Kobe, Japan

**Keywords:** atherosclerosis, cerebral arteries, hyperlipidemia, hypertension, rabbits

## Abstract

**Background:**

Intracranial atherosclerosis (ICAS) is one of the most common causes of ischemic stroke, but there are few animal models that can recapitulate its pathological features. In this study, we examined ICAS pathological features and anatomic distributions using three types of hyperlipidemic rabbit models. We also investigated the effect of different lipoprotein profiles and hypertension on ICAS.

**Materials and Methods:**

We examined Watanabe heritable hyperlipidemic (WHHL) rabbits, apoE knockout (KO) rabbits and wild-type rabbits (WT) fed a cholesterol diet, in addition to WT rabbits fed a standard diet as a control. The whole brain was dissected and embedded in paraffin. Serial sections were stained with either hematoxylin/eosin or elastica van Gieson, or immunohistochemically stained with monoclonal antibodies against macrophages and smooth muscle cells. We investigated (1) the presence of cerebral atherosclerosis; (2) the lesion locations in the cerebral arteries; (3) the degree of lumen stenosis; (4) pathological features and cellular components of the lesions in these rabbits; and (5) whether hypertension affects ICAS.

**Results:**

ICAS was detected in apoE and WHHL rabbits, but not in WT rabbits. Compared with apoE KO rabbits, WHHL rabbits had greater ICAS. The lesions of cerebral atherosclerosis were mainly distributed at the bifurcations of the posterior cerebral artery, basilar artery and vertebral artery, and they were basically characterized by smooth muscle cells and extracellular matrix with few macrophages. The extent of the ICAS in WHHL rabbits was significantly increased by hypertension.

**Conclusions:**

ICAS was detected in WHHL and apoE KO rabbits, and occurred in specific locations in the cerebral arteries. Hypertension promotes the development of ICAS in the setting of hypercholesterolemia.

## Introduction

Cerebral atherosclerosis is widely considered the most important cause of ischemic stroke. The prevalence of cerebral atherosclerosis is as high as 50% of ischemic stroke patients in different populations (1–3). However, the diagnostic and therapeutic strategies for cerebral atherosclerosis vary due to a poor understanding of the molecular mechanisms of its pathogenesis.

It is well known that cerebral atherosclerosis develops later and exhibits more variability among different populations than aortic and coronary atherosclerosis (4, 5). Human autopsy studies suggested that the extent of cerebral atherosclerosis in Asians is greater than in Caucasians (6). Cerebral atherosclerosis generally occurs in relatively large cerebral arteries, such as the internal carotid artery and middle cerebral artery, and the lesions are pathologically characterized by stable features and rarely exhibit advanced complications, such as calcification and intraplaque hemorrhage, which are often noted in coronary atherosclerosis (4, 7). Although it is not known why the features of intracranial atherosclerosis differ from other extracranial atherosclerosis, the specific location of the skull may determine the lesion pathology even with the same risk factors (8). Clinical studies revealed that high plasma levels of low-density lipoprotein (LDL) and low plasma levels of HDL-cholesterol are closely correlated with the severity of cerebral atherosclerotic stenosis (9–11). In addition, a postmortem study demonstrated that cerebral atherosclerosisis present in unborn fetuses of hypercholesterolemic mothers (12, 13). Furthermore, hypertension increases the risk of cerebral atherosclerosis in patients with high levels of LDL cholesterol.

As the pathological features and pathogenesis of cerebral atherosclerosis may differ from those of extracranial atherosclerosis, it is necessary to develop appropriate animal models. The cerebral arteries unlike extracranial arteries are generally resistant to hypercholesterolemia in experimental animals (14–18); therefore, there are few experimental animal models that can be used for the study of cerebral atherosclerosis. Rabbits are one of the best animal models for the study of human lipid metabolism and atherosclerosis because they are sensitive to a cholesterol diet and rapidly develop hypercholesterolemia and atherosclerosis (19, 20). In cholesterol-fed rabbits, aortic atherosclerosis is easy to induce and analyze; therefore, almost all studies using cholesterol-fed rabbits focused on aortic atherosclerosis, with a few studies on coronary atherosclerosis, whereas cerebral atherosclerosis was not fully investigated (14, 15, 21). WHHL rabbits are often used for studies on atherosclerosis because they are genetically deficient in LDL receptor functions, and spontaneously develop hypercholesterolemia and atherosclerosis even on a standard diet (22). In addition, several knockout (KO) rabbits have been established recently and applied for the study of atherosclerosis, including apoE, LDL receptor and cholesteryl ester transfer protein (23–25). However, pathological investigations of cerebral atherosclerosis using these rabbit models have not been systemically performed. We conducted the current study using three kinds of hypercholesterolemic rabbits, cholesterol-fed wild-type (WT), apoE KO and WHHL rabbits, to examine the severity of cerebral atherosclerosis. We performed this study with the following questions in mind: (i) Does cerebral atherosclerosis occur in these hypercholesterolemic rabbits? (ii) What cerebral arteries are involved? (iii) What are the pathological features? As hypertension is another major risk factor for the development of cerebral atherosclerosis in addition to hypercholesterolemia, we also examined cerebral atherosclerosis in hypertensive WHHL rabbits (26).

## Materials and Methods

### Rabbits

In this study, we pathologically examined cerebral atherosclerosis in rabbits. For this purpose, we used three kinds of hypercholesterolemia rabbits along with normal control rabbits. ApoE KO and WT NZW rabbits aged 7 months (6 males and females for both groups) were fed a cholesterol diet for 16 weeks. The cholesterol diet used for this experiment was composed of the normal standard diet (17% protein, 4% fat, and 14% crude fiber) (CLEA Japan, Inc, Tokyo, Japan), which was supplemented with 0.3% cholesterol and 3% soybean oil, which did not affect the body weight (27). These two models exhibited hypercholesterolemia due to the accumulation of plasma remnant lipoproteins (β-VLDLs) (28). In addition, we used male WHHL rabbits aged 20 ± 8 months (ranging from 12 to 31 months) which are genetically deficient in LDL receptor functions and exhibited high levels of plasma LDLs, resembling human familial hypercholesterolemia (28). Using these three models, we investigated whether different types of hypercholesterolemia exert different effects on the development of cerebral atherosclerosis. Furthermore, we examined cerebral atherosclerosis in hypertensive WHHL rabbits induced by 1K1C (surgical removal of the left kidney and partial ligation of the right renal artery) methods, as reported in previous studies (26, 27). All animal experiments were performed according to the approval of the Animal Care Committee of the University of Yamanashi and conformed to the Guide for the Care and Use of Laboratory Animals published by the US National Institutes of Health.

### Analysis of Plasma Lipids and Lipoprotein Profiles

After fasting for 16 hours, blood was collected from rabbits, as described previously (28). In brief, blood was collected through a central auricular artery and kept in microtubes pre-loaded with EDTA and aprotinin. EDTA-plasma was obtained after centrifuging at 4000rpm for 20min at 4°C. Plasma total cholesterol (TC), triglycerides (TG), and high-density lipoprotein-cholesterol (HDL-C) were measured using commercial assay kits (Wake Pure Chemical Industries, Ltd, Osaka, Japan). Plasma lipoprotein profiles were analyzed by the method of high-performance liquid chromatography(HPLC) described previously (29). Plasma samples (4μl) were analyzed by HPLC on a gel filtration column by Skylight Biotech (Akita, Japan).

### Vascular Casting

To map the lesion distribution in the cerebral vascular system, we first performed rabbit brain vascular casting. For this purpose, three NZW rabbits were sacrificed by intravenous injection of an overdose of sodium pentobarbital solution (5ml per rabbit). A longitudinal incision was made in the middle of the neck to expose the common carotid artery. Then, the proximal part of the carotid common artery was ligated and a “V” shape surgical incision was made 5cm away from the proximal ligation, through which one 16-gauge catheter (509083, Sherwood Medical, ST. Louis, MO) was inserted into the bilateral common carotid arteries and fixed stably with sutures. After cutting the internal jugular vein, rabbits were perfused with saline solution *via* the common carotid artery until the liquid from the internal jugular vein became clear.

The casting solution was prepared according to the manufacturer’s protocol. Briefly, red pigment (07350, Polysciences, Inc., PA) was added in the amount of 2% to Base solution A and stirred vigorously for intensive mixing. Then, the solution was divided into two equal parts and half was prepared with 40% of Catalyst (02608, Polysciences, Inc., PA), whereas the remaining solution was mixed with Promoter C (02610, Polysciences, Inc., PA) completely. Afterward, the casting agent solution was injected at approximately 8-10ml/min until the casting agent was visualized in the vertebral arteries. The tissue was corroded in 20% KOH solution (168-21815, Wako Pure Chemical Corporation, Osaka, Japan) for approximately 48h at 4°C. Vascular casting was collected, and the peripheral tissues were washed off carefully and kept at 37°C for drying.

### Dissecting Brains

**Figure 1:**
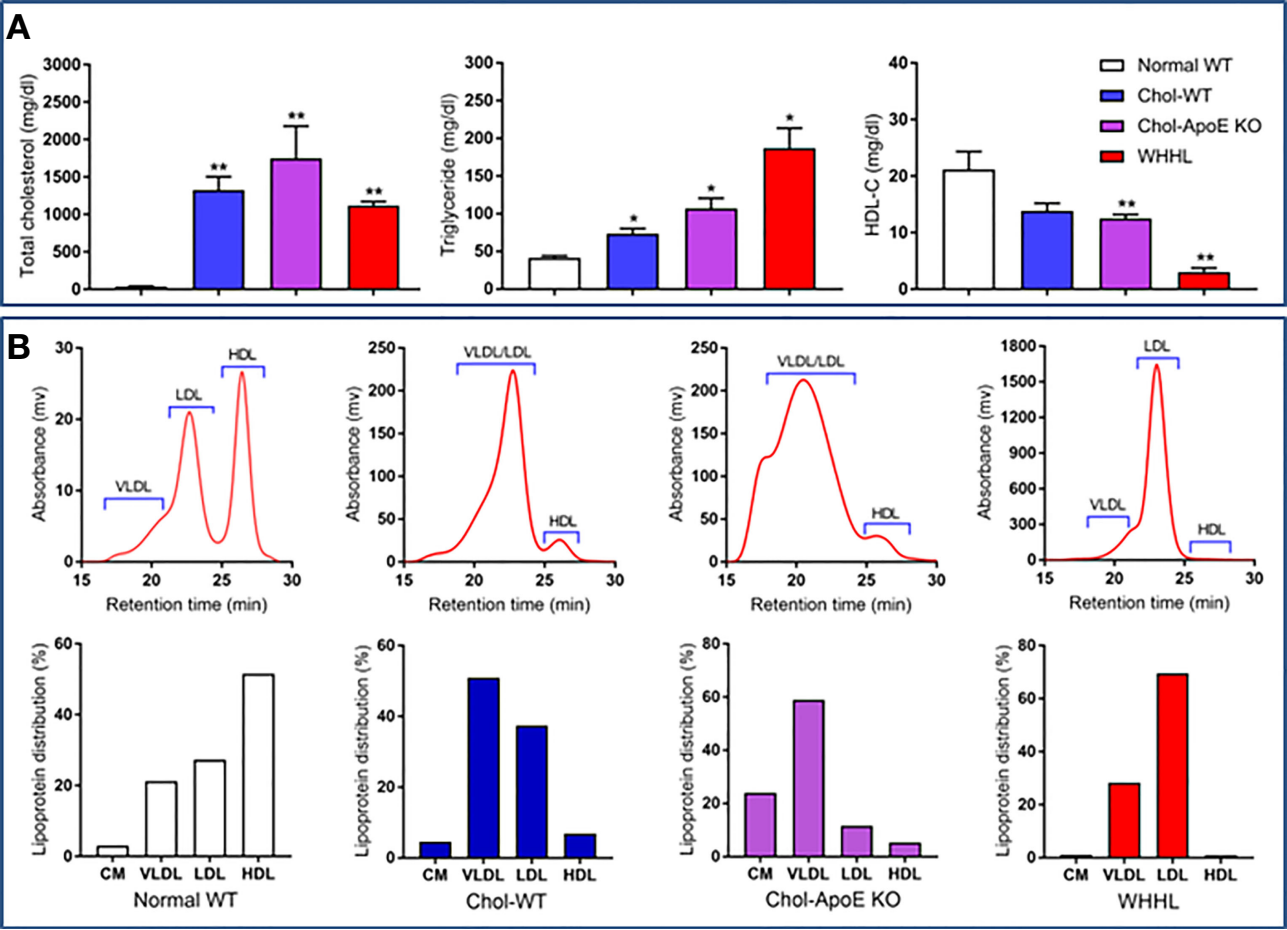
Plasma lipids **(A)** and lipoprotein profiles **(B)**. Plasma lipids including total cholesterol (TC), triglycerides (TG), and high-density lipoprotein cholesterol (HDL-C) were measured in different rabbits fasted overnight. All animals were males and n=6 for each group and the data are expressed as the mean ± SEM. Plasma lipoproteins profiles were analyzed by HPLC gel filtration and representative data are shown (**B**, top panel). Lipoprotein distribution (%) was calculated using cholesterol values of each fraction divided by total values (**B**, bottom panel). *p<0.05, **p<0.01 vs. normal WT.

The whole brain was isolated and fixed in 10% buffered formalin solution. After fixing, the whole brain was dissected into 10 slices (approximately 10mm thick) at intervals of 3mm in the region of posterior circulation and 6mm in the region of anterior circulation to prepare paraffin specimens (**S-Figure 1**). All slices were placed in cassettes rostral side down for paraffin embedding.

### Pathological Analysis of Cerebral Atherosclerosis

To analyze the pathological characteristics of cerebral atherosclerotic lesions, specimens were cut into 3-μm serial sections and used for hematoxylin and eosin (HE) and elastica van Gieson (EVG) staining. The lesions were also immunohistochemically stained with monoclonal antibodies against rabbit macrophage RAM11 (M0633, Dako Co., Carpinteria, CA) and smooth muscle α-actin HHF35 (M0635, Dako Co., Carpinteria, CA). HE-stained specimens were first examined under a light microscope to find lesions along with evaluations of the lesion location and pathological features. The lumen stenostic rate was measured using EVG-stained specimens and calculated as atherosclerotic lesion area/lumen area x % as we did for coronary atherosclerosis (19). Lesional cellular components were investigated using immunohistochemically stained specimens. All images were incorporated into a digital camera and analyzed using the image analysis system WinRooF V6.4.0 (Mitani Co, Tokyo, Japan).

### Statistical Analysis

All data were expressed as the mean ± SEM. Data were analyzed using GraphPad Prism 7.0 (GraphPad Software, San Diego, CA). The Shapiro-Wilk test was used to verify the normal distribution of all data. The Student’s *t*-test and ANOVA were used. *P*-values less than 0.05 were considered significant.

## Results

As shown in **Figure 1A**, all three types of hyperlipidemic rabbits exhibited high plasma TC and TG levels, with lower HDL-C levels than WT rabbits. Moreover, lipoprotein profiles differed among the three groups (**Figure 1B**, top panel). In WT rabbits on a standard diet, plasma cholesterol was mainly distributed in VLDL/LDL and HDL particles. On a cholesterol diet, both VLDLs and LDLs markedly increased in WT whereas chylomicrons and VLDLs were mainly elevated in apoE KO rabbits. In WHHL rabbits; however, LDL particles increased remarkably (**Figure 1B**, bottom panel) as reported (28).

**Figure 2:**
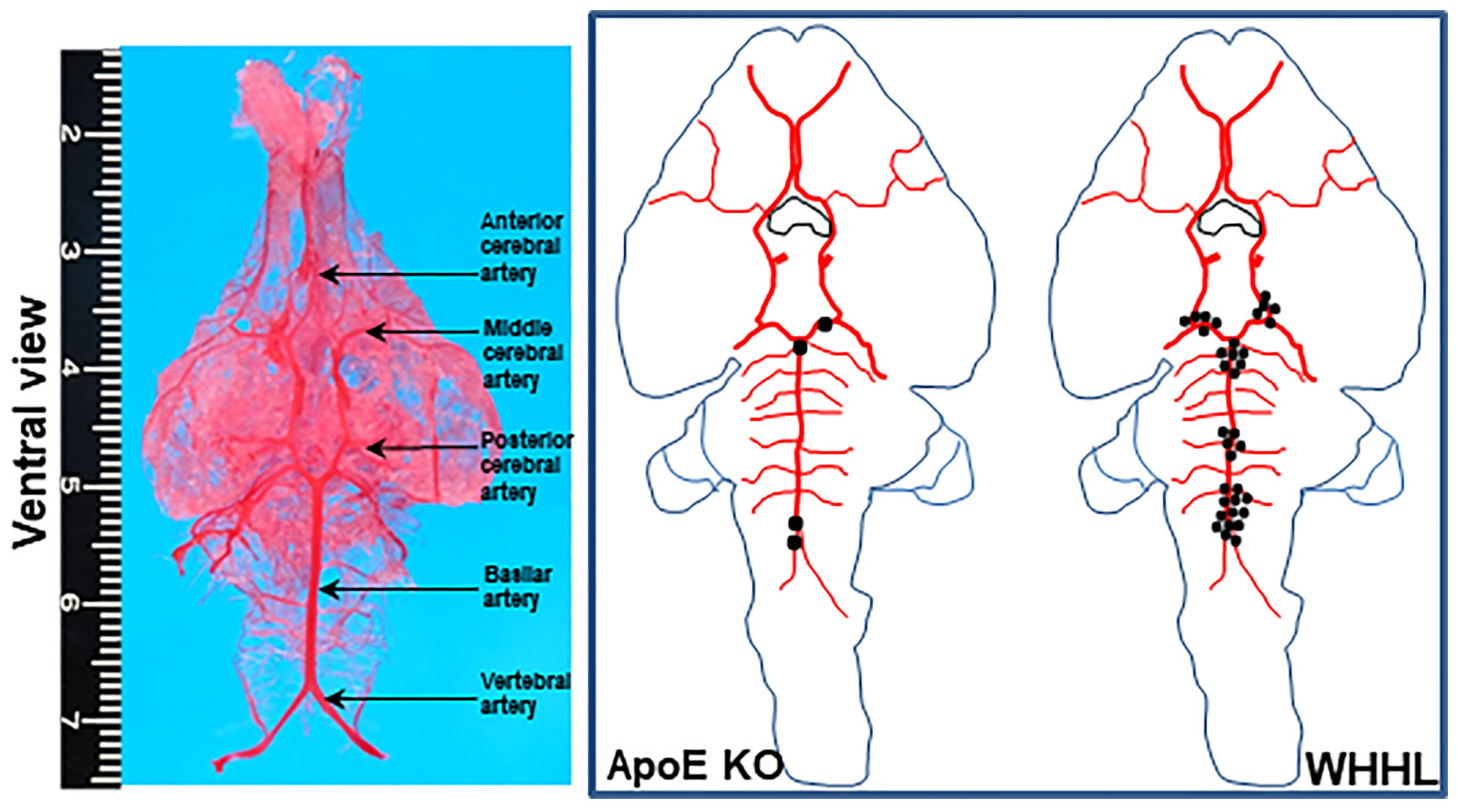
Vascular casting of cerebral arteries (left) and schematic mapping of the distribution of all intracranial atherosclerosis in all sections (12 sections/each animal) of apoE KO (n=12) and WHHL rabbits (n=15)(right). Hematoxylin-eosin stained-sections were used to observe the arteries with lesions. Each spot indicates one lesion site detected under the light microscopic observations. All lesions were distributed in PCA, BA, and VA.

To investigate the cerebral arterial pathology, we first performed cerebrovascular casting in order to illustrate the anatomic distribution of all arteries in the brain using normal WT rabbits (**Figure 2**). According to these artery distributions, we were able to visualize the five major branches: anterior cerebral artery, middle cerebral artery, posterior cerebral artery (PCA), basilar artery (BA) and vertebral artery (VA), with average diameters of 169 ± 12, 169 ± 11, 288 ± 39, 300 ± 25 and 316 ± 22μm, respectively. After thorough examinations of the whole brain arteries under a light microscope, we did not find any cerebral atherosclerosis in WT rabbits either on a standard or cholesterol diet for 16 weeks. However, we observed foam cell accumulation in the choroid plexus (**S-Figure 2**).

**Figure 3:**
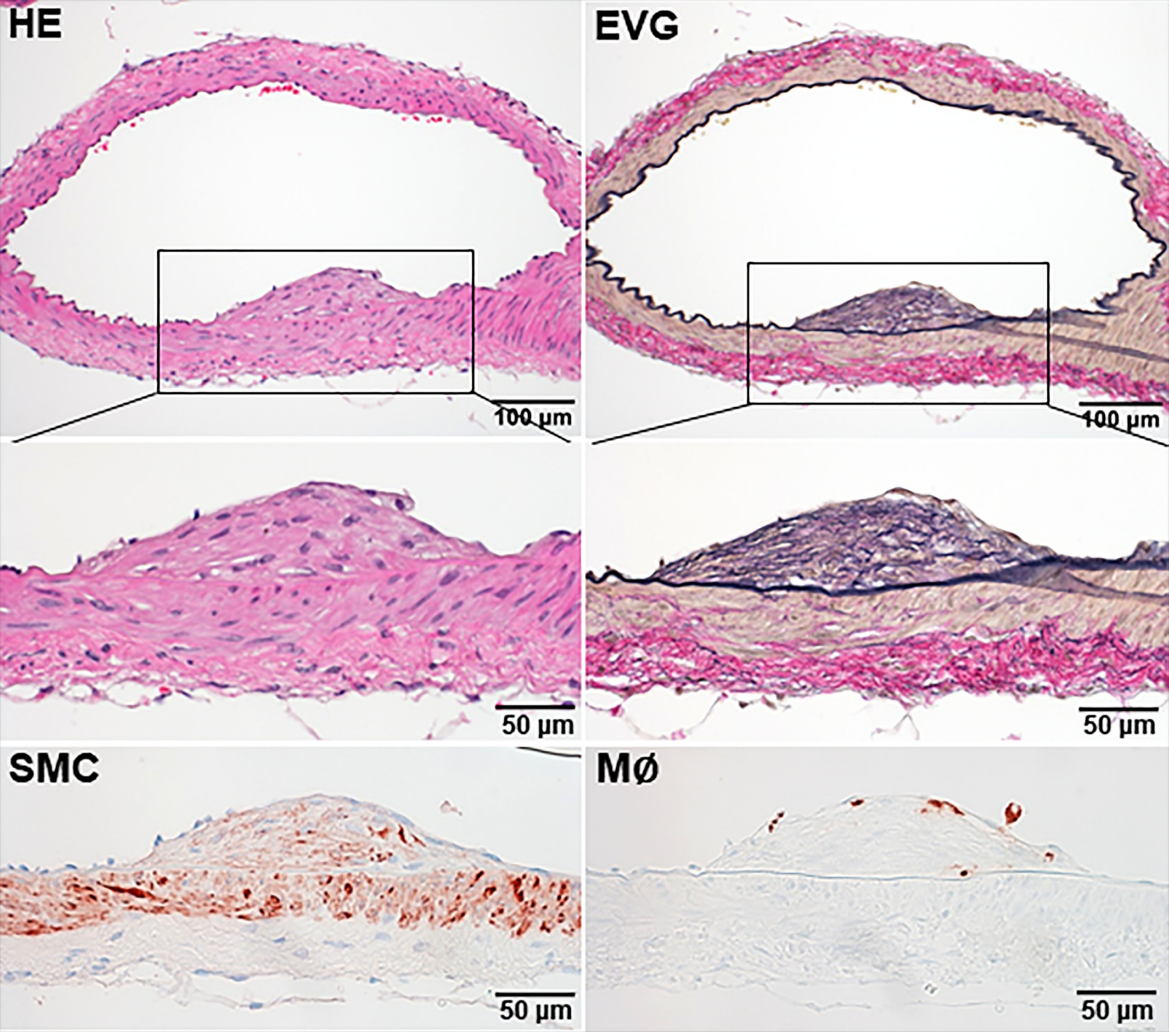
Histological features of intracranial atherosclerosis of a cholesterol diet-fed apoE KO rabbit. Paraffin-embedded specimens were stained with Hematoxylin-eosin (HE) and elastica van Gieson (EVG) or immunohistochemically stained with antibodies against either macrophages (M∅) or smooth muscle cells (SMC). A small intimal lesion sized 250μm in length was observed in PCA and mainly composed of fibrous tissue with a few smooth muscle cells and macrophages. This kind of lesions was only observed in 3 rabbits among 12 apoE KO rabbits.

We then examined apoE KO rabbits fed a cholesterol diet because apoE KO rabbits on a normal regular diet exhibited mild hyperlipidemia, which is not atherogenic (28). Among 12 apoE KO rabbits fed a cholesterol diet, 3 (2 males and 1 female) exhibited cerebral atherosclerosis; two lesions were in the confluence of VA-BA and one was in the BA-PCA bifurcation, suggesting that cerebral atherosclerosis developed in these branches, although the incidence was markedly low (**Figure 2**). Histological examinations of these lesions revealed 6%~13% lumen stenosis. On immunohistochemical staining, the lesions were mainly composed of smooth muscle cells and extracellular matrix with few macrophages (**Figure 3**). Similar to WT rabbits, foam cell accumulation in the choroid plexus was often observed (**S-Figure 2**).

**Figure 4:**
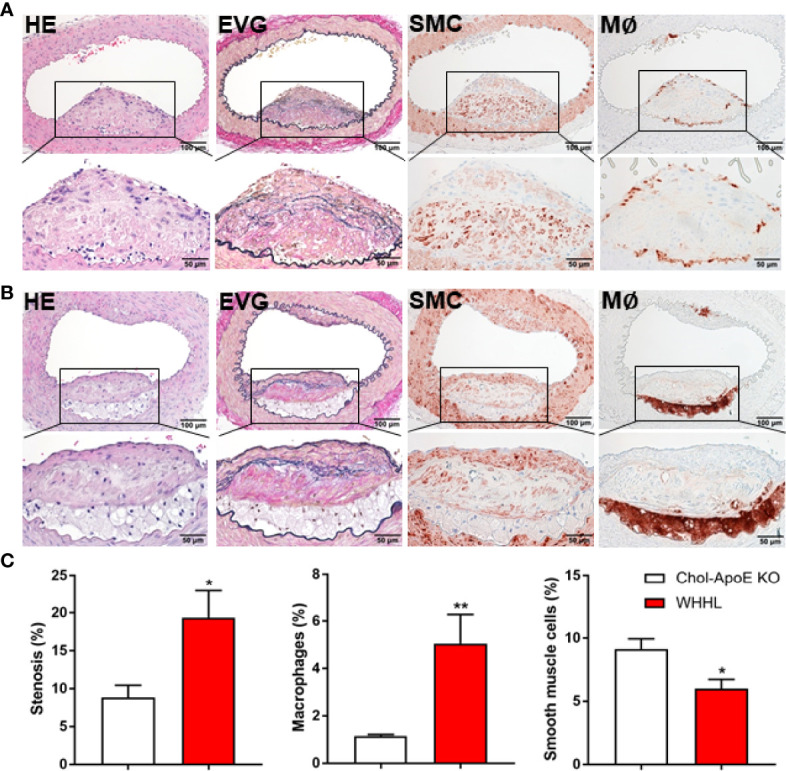
Representative micrographs of intracranial atherosclerosis of WHHL rabbits fed on a normal regular diet. Among 15 rabbits, 13 rabbits showed various degree of the cerebral lesions. Most intimal lesions were characterized by fibrosis intermingled with smooth muscle cells in the center and few macrophages on the top [lesions of the basilar **(A)**]. Two animals showed intimal lesions rich in macrophages at the bottom of the lesion **(B)**. Lumen stenosis rate, macrophages and smooth muscle cells in the arteries with the lesions of all sections (n=4 for apoE KO and n=32 for WHHL) were quantified, compared, and expressed as % **(C)**. *p<0.05, **p<0.01 vs. chol-apoE KO. Actual areas were shown in **Supplemental Data** (**S-Table 5**).

In contrast to WT and apoE KO rabbits fed a cholesterol diet, 87% (13/15) of WHHL rabbits exhibited differing degrees of cerebral atherosclerosis in 3 major branches: PCA (43%), BA (73%) and VA (26%), but not in other branches (**Figure 2**), whereas two rabbits (aged 14 and 27 months) had no lesions. These cerebral lesions caused differing degrees of lumen stenosis, ranging from 2% to 82%. Immunohistochemical staining demonstrated that almost all lesions were rich in smooth muscle cells and extracellular matrix (**Figure 4A**), whereas few lesions contained more macrophage-derived foam cells (**Figure 4B**). Quantitation of the stenosis rate and cellular components of the lesions revealed that cerebral atherosclerosis in WHHL rabbits was severer and the lesions contained more macrophages than that of apoE KO rabbits (**Figure 4C**). In addition, there was notable foam cell accumulation and cholesterol crystal in the choroid plexus (**S-Figure 2**).

**Figure 5:**
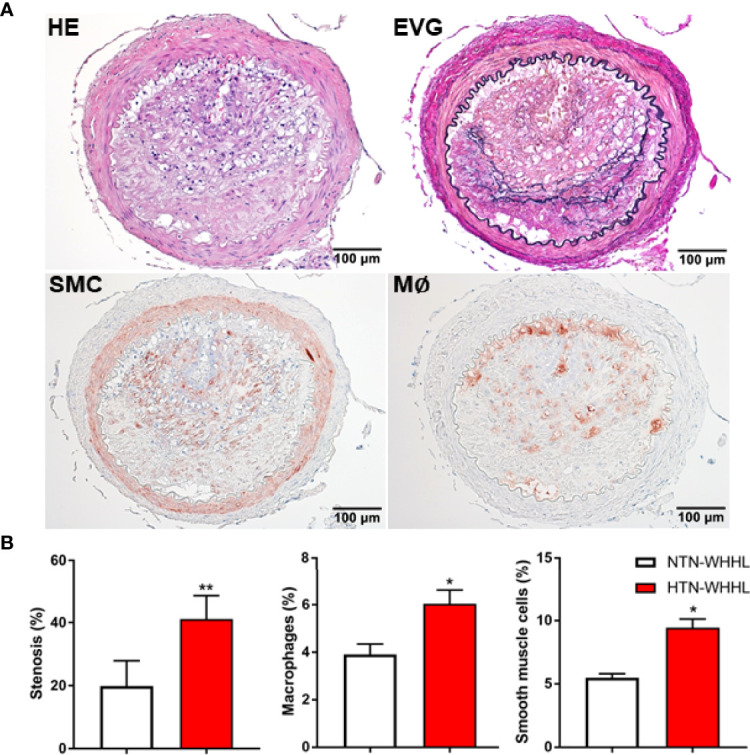
Representative micrographs of intracranial atherosclerosis of WHHL rabbits with renovascular hypertension **(A)**. Renovascular hypertension was induced at 8 mon and systolic and diastolic pressure was maintained at 160 mm Hg (110 mm Hg in control) and 110 mm Hg (100 mm Hg in control) for 12 mon(25). Normotensive group was subjected to a sham operation and used as controls. Serial sections were stained with hematoxylin-eosin (HE) and elastica van Gieson (EVG) or immunohistochemically stained with antibodies against either macrophage (M∅) or smooth muscle cells (SMC). The lesions of the vertebral arteries of a hypertensive WHHL rabbit are almost occlusive and rich in macrophages. Lumen stenosis rate, macrophages and smooth muscle cells in the lesions (n= 14 for hypertensive and n=4 for normotensive) were quantified and expressed as % **(B)**. *p<0.05, **p<0.01 vs. normotensive. Actual areas were shown in **Supplemental Data** (**S-Table 6**).

As 87% of WHHL rabbits exhibited cerebral atherosclerosis, we further investigated the influence of renovascular hypertension on lesion formation in these rabbits. As shown in **Figure 5**, compared with normotensive WHHL rabbits, all hypertensive WHHL rabbits exhibited greater cerebral atherosclerosis in VA (5/5), BA (2/5), PCA (3/5), which led to a 2-fold increase of the lumen stenosis (*p*<0.01) due to significantly increased macrophages and smooth muscle cells in the lesions of hypertensive WHHL rabbits (*p*<0.05).

## Discussion

In the current study, we performed a systemic examination of cerebral atherosclerosis using three hypercholesterolemic rabbits with different lipoprotein profiles. Similar to the previous study (14), cholesterol-diet feeding alone failed to induce cerebral atherosclerosis in WT rabbits, suggesting that cerebral arteries are generally resistant to hypercholesterolemia compared with aortas and coronary arteries. Next, we examined apoE KO rabbits, which have higher TC and TG levels than WT rabbits, and more atherosclerosis in the aorta and coronary arteries (25). ApoE is a glycoprotein synthesized mainly in the liver and brain, which functions as a ligand of LDL receptors and lipoprotein receptor-related proteins for the clearance of chylomicrons and VLDL remnants, in addition to maintenance of the blood-brain barrier and regulation of brain inflammation and atherosclerosis (30–32).

However, only a few apoE KO rabbits exhibited tiny lesions in the cerebral arteries, which is consistent with other studies (33, 34). This result suggests that high levels of cholesteryl ester-rich remnant lipoproteins were not highly atherogenic to cerebral arteries compared with extracranial arteries regardless of the absence of apoE in these particles, although apoE deficiency significantly increases the development of both aortic and coronary atherosclerosis in these rabbits (Niimi et al. unpublished data).

Furthermore, we analyzed WHHL rabbits in which aortic and coronary atherosclerosis started to form as early as 3 months. Although WHHL rabbits have been extensively studied, the incidence and distribution of cerebral atherosclerosis was not clarified. Differing from cholesterol-fed WT and apoE KO rabbits, WHHL rabbits had high levels of plasma LDLs, similar to human familiar hypercholesterolemia. In the current study, WHHL rabbits aged 12 to 31 months exhibited a higher incidence of cerebral atherosclerosis (87%) and severe stenosis than apoE KO rabbits, suggesting that LDLs are more atherogenic than β-VLDLs in terms of cerebral atherosclerosis, which is consistent with the human imaging and pathological observations (7, 35, 36). Furthermore, 72% of the lesions in WHHL rabbits were distributed at the bifurcations of the arteries of posterior cerebral circulation. It should be pointed out; however, human ICAS is predominantly present in the anterior circulation such as the circle of Willis (7, 35, 36). The discrepancy between rabbits and humans in terms of ICAS locations is currently unknown but may be owing to several factors. For example, the brain circulation of “recumbent” rabbits may be different from that of “upright” humans including blood pressure, hemodynamics, and responsiveness of the arteries. Furthermore, humans usually have more risk factors and longer time of the lesion progression than experimental rabbits. In line with these possibilities, it may also help explain why in humans, carotid arteries are often affected by atherosclerosis whereas in rabbits, these arteries are usually spared as we did not find any lesions. However, when we expressed human apo(a) gene in transgenic rabbits, we can observe carotid atherosclerosis (37), suggesting that specific risk factors are required for the lesion formation in rabbits.

In spite of this, the lesions of cerebral atherosclerosis in WHHL rabbits are structurally stable, and characterized by smooth muscle cells and extracellular matrix unlike the foam cell-rich lesions observed in aortic atherosclerosis in these rabbits. These features are similar to those of human intracranial atherosclerosis, which comprises more stable lesions than extracranial atherosclerosis (4, 6, 7). However, the severity of intracranial atherosclerosis in both WHHL and apoE KO rabbits was lower than that of extracranial atherosclerosis in the same animals. For example, we compared the stenosis rate of the coronary and cerebral atherosclerosis in WHHL and apoE KO rabbits in the current study and found that average coronary stenosis rate was much higher than that of cerebral lesions (89 ± 3.7% and 63.8 ± 17% in coronary arteries of WHHL and apoE KO respectively) in the same animals. Although why cerebral arteries (compared with coronary arteries and aortas) are resistant to atherosclerosis remains unclear, several possible factors may be involved. Cerebral arteries are characterized by having the tight junctions of endothelial cells, and a thick subendothelial basement membrane and internal elastic lamina (14, 38). Such structures constitute the first barrier to prevent the efflux of plasma atherogenic lipoproteins into the subendothelial space to initiate atherosclerosis. We further compared the structural differences of the major cerebral arteries with coronary arteries of WT rabbits (**S-Figure 3** and **S-Table 4**). The diameter of the right and left coronary arteries is approximately 2- and 4-fold larger, respectively, than the basilar and vertebral arteries in the same WT rabbits, but the medial layers were 3 ~ 4-fold larger (3–4 layers in cerebral vs 12–13 layers in coronary arteries). In addition, unlike coronary arteries, cerebral arteries lack an explicit external elastic lamina (**S-Figure 3**). These distinct histological features may also be involved in the resistance to cerebral atherosclerosis. Moreover, blood flow and blood pressure in the skull likely differ from those at extracranial sites, thereby affecting the vascular functions. Hypertension is one of the essential factors in the development and progression of intracranial atherosclerosis (4). As the risk of cerebral atherosclerosis is significantly increased by hypertension in the setting of hypercholesterolemia (18, 21, 39), we examined the effects of renovascular hypertension on the development of cerebral atherosclerosis in WHHL rabbits (26). A marked increase in the incidence and degree of stenosis due to significantly increased macrophages in the lesions, were observed in hypertensive WHHL rabbits, strengthening the notion that hypertension accelerates the development of cerebral atherosclerosis. High blood pressure may cause endothelial damage and lead to the retention of plasma atherogenic lipoproteins in the subintima, thereby exacerbating cerebral atherosclerosis in the presence of hypercholesterolemia (15, 21, 40). Hypertension not only increases the incidence, but also the lesion size with more macrophages in the cerebral arteries. This also suggests that this hypertensive WHHL rabbit model is a useful tool to examine cerebral atherosclerosis.

In conclusion, cerebral atherosclerosis can be generated in both apoE KO and WHHL rabbits, but the latter exhibited more lesions. The high incidence and severity of cerebral atherosclerosis in WHHL rabbits suggest that LDLs are more atherogenic than remnant lipoproteins. Furthermore, hypertension promotes the development of cerebral atherosclerosis in the setting of hypercholesterolemia.

## Data Availability Statement

The original contributions presented in the study are included in the article/**Supplementary Material**. Further inquiries can be directed to the corresponding author.

## Ethics Statement

The animal study was reviewed and approved by the Animal Care Committee of the University of Yamanashi.

## Author Contributions

JF designed the experiments and provided the resources. XT, MN and HZ performed the experiments and analyzed the data. LC, YC and HY bred animals and participated in experiments. XT and JF wrote the manuscript. All authors contributed to the article and approved the submitted version.

## Funding

This work was supported in part by JSPS KAKENHI (JP17K08783 and JP15H04718), the National Natural Science Foundation of China (No. 81941001 and 82100482), the JSPS-CAS Bilateral Joint Research Program (JPJSBP 120187204) and Innovation team program supported by Guangdong Province (2020KCXTD038). XT was a recipient of the China Scholarship Council.

## Conflict of Interest

The authors declare that the research was conducted in the absence of any commercial or financial relationships that could be construed as a potential conflict of interest.

## Publisher’s Note

All claims expressed in this article are solely those of the authors and do not necessarily represent those of their affiliated organizations, or those of the publisher, the editors and the reviewers. Any product that may be evaluated in this article, or claim that may be made by its manufacturer, is not guaranteed or endorsed by the publisher.

## Abbreviations

Apo, Apolipoprotein; BA, Basilar artery; EVG, Elastica van Gieson; HDL-C, High-density lipoprotein-cholesterol; HE, Hematoxylin and eosin; ICAS, Intracranial atherosclerosis; KO, Knockout; LDL, Low-density lipoprotein; PCA, Posterior cerebral artery; TG, Triglycerides; VA, Vertebral artery; VLDL, Very low-density lipoprotein; WHHL, Watanabe heritable hyperlipidemic; WT, Wild-type.
