# Supplementary material for: Pathological Investigations of Intracranial Atherosclerosis Using Multiple Hypercholesterolemic Rabbit Models
Source: Front Endocrinol (Lausanne). 2022 May 27;13:834207. doi: 10.3389/fendo.2022.834207 (PMC9196249; doi:10.3389/fendo.2022.834207)
Supplement: Supplementary file 1 [file DataSheet_1.pdf]

S-Table 1. Examinations of cerebral atherosclerosis in WT and apoE KO rabbits fed a cholesterol diet

Groups	No.	PCA	BA	VA
**WT** (male,7 mon)	**1**	ud	ud	ud
	**2**	ud	ud	ud
	**3**	ud	ud	ud
	**4**	ud	ud	ud
	**5**	ud	ud	ud
	**6**	ud	ud	ud
**WT** (female,7 mon)	**1**	ud	ud	ud
	**2**	ud	ud	ud
	**3**	ud	ud	ud
	**4**	ud	ud	ud
	**5**	ud	ud	ud
	**6**	ud	ud	ud
**ApoE KO** (male,7 mon)	**1**	ud	ud	ud
	**2**	ud	1	ud
	**3**	ud	ud	ud
	**4**	ud	ud	ud
	**5**	1	ud	1
	**6**	ud	ud	ud
**ApoE KO** (female,7 mon)	**1**	ud	ud	ud
	**2**	ud	ud	ud
	**3**	ud	ud	ud
	**4**	ud	ud	1
	**5**	ud	ud	ud
	**6**	ud	ud	ud

The number of lesions from all sections was counted. ud indicates “undetected”. PCA: posterior cerebral artery; BA: basilar artery; VA: vertebral artery.

S-Table 2. Examinations of the incidence of cerebral atherosclerosis in WHHL

No.	Gender and age(month)	PCA	BA	VA
**1**	Male, 12m	ud	2	1
**2**	Male, 14m	ud	1	ud
**3**	Male, 14 m	ud	2	ud
**4**	Male, 14 m	ud	ud	ud
**5**	Male, 15 m	ud	4	ud
**6**	Male, 15 m	1	1	2
**7**	Male, 15 m	1	2	1
**8**	Male, 17 m	2	2	ud
**9**	Male, 17 m	1	ud	ud
**10**	Male, 22 m	ud	1	ud
**11**	Male, 24 m	1	ud	ud
**12**	Male, 25 m	1	1	ud
**13**	Male, 27 m	ud	ud	ud
**14**	Male, 28 m	ud	1	ud
**15**	Male, 31 m	1	2	1

The number of lesions from all sections was counted. ud indicates “undetected”. PCA: posterior cerebral artery; BA: basilar artery; VA: vertebral artery.

S-Table 3.Examination of cerebral atherosclerosis in hypertensive and normotensive WHHL rabbits

Group	No.	Gender and age(month)	PCA	BA	VA
Hypertensive	**1**	Female, 18 m	1	ud	1
	**2**	Female, 20 m	ud	ud	1
	**3**	Male, 21 m	2	3	1
	**4**	Female, 22 m	ud	ud	1
	**5**	Female, 22 m	2	1	1
Normotensive	**1**	Female, 22 m	ud	ud	2
	**2**	Female, 22 m	ud	ud	ud
	**3**	Female, 22 m	ud	1	ud
	**4**	Female, 22 m	ud	ud	ud
	**5**	Female, 22 m	ud	ud	1

The number of lesions from all sections was counted. ud indicates “undetected”. PCA: posterior cerebral artery; BA: basilar artery; VA: vertebral artery.

S-Table 4. Comparison of coronary and cerebral histological features

	Cerebral artery	Coronary artery
**Thickness of IEL (μm)**	5.7±2.8	4.7±0.8
**Layers of SMCs in media**	4±1	13±1
**Layers of elastic fibers in media (diameter=400μm)**	4±1	13±1
**Layers of elastic fibers in media (diameter=100μm)**	4±1	4±1
**External elastic lamina**	Absent	Present

IEL: internal elastic lamina; SMC: smooth muscle cells
The thickness of IEL was assessed through randomly selected four high power fieldsand averaged. All values are expressed as the mean±SD.

S-Table 5. Examinations of cerebral atherosclerosis in chol-ApoE KO and WHHL rabbits

Group	Lesion area (μm^2)	Macrophage area (μm^2)	SMC area (μm^2)
**Chol-ApoE KO**	12612±2174	82±23	1241±116
**WHHL**	49855±1745*	696±87**	1867±213*

Data were expressed as mean±SE. *p<0.05, **p<0.01 vs Chol-ApoE KO group.

S-Table 6. Examinations of cerebral atherosclerosis in hypertensive and normotensive WHHL rabbits

Group	Lesion area (μm^2)	Macrophage area (μm^2)	SMC area (μm^2)
**Normotensive**	49728±9202	448±98	2620±189
**Hypertensive**	94764±6210**	1344±214*	4025±255*

Data were expressed as mean±SE. *p<0.05, **p<0.01 vs normotensive group.

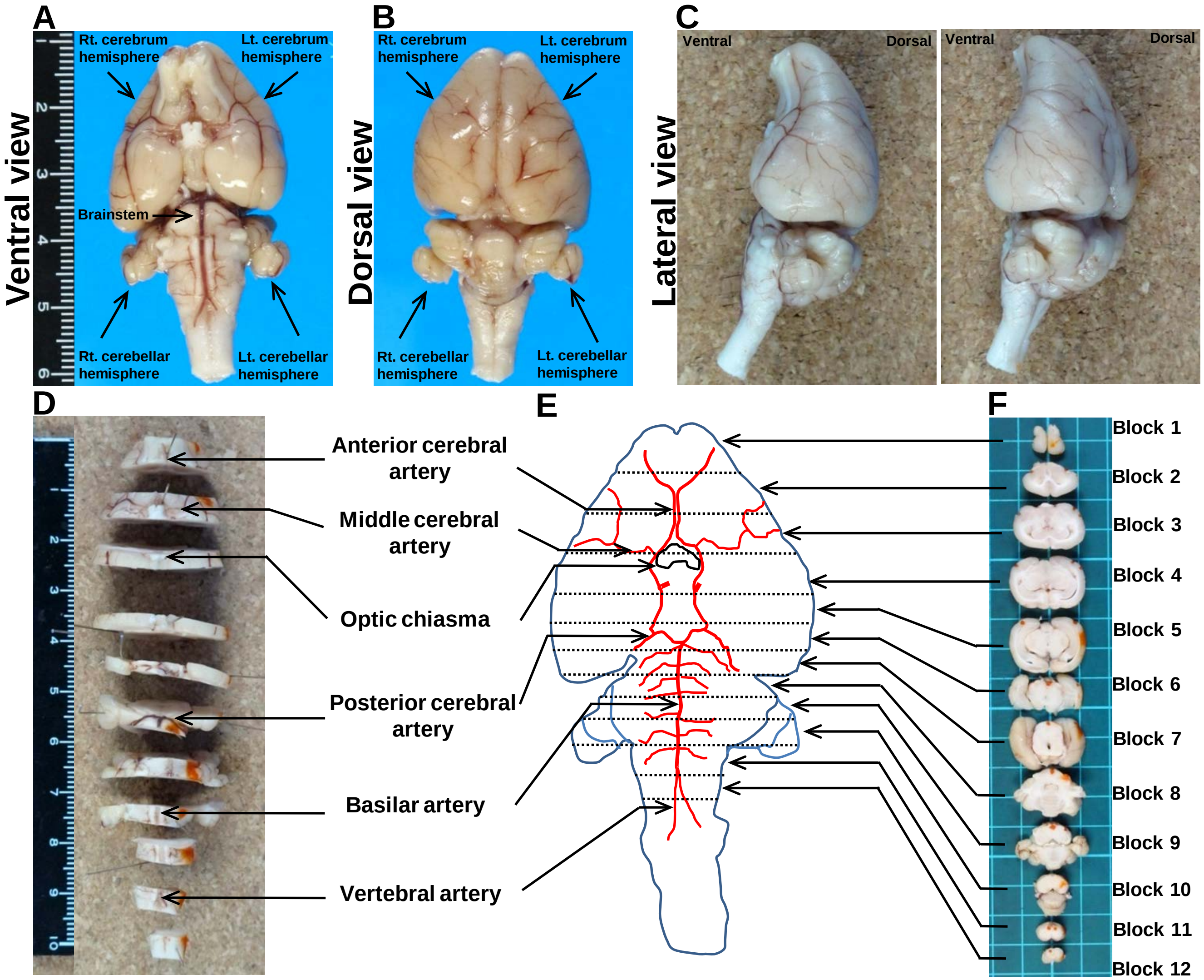

S-Figure 1. The methods for dissecting rabbit brains. The rabbit brain was fixed in 10% formalin and viewed from different angles: ventral (A) and dorsal (B) and lateral view (C, D). The whole brain is sliced into 12 segments at appropriately 6 mm intervals for cutting blocks 1-4 and 3 mm for cutting blocks 5-12 (D). The arterial sites in each block are matched with vascular mapping (E and F).

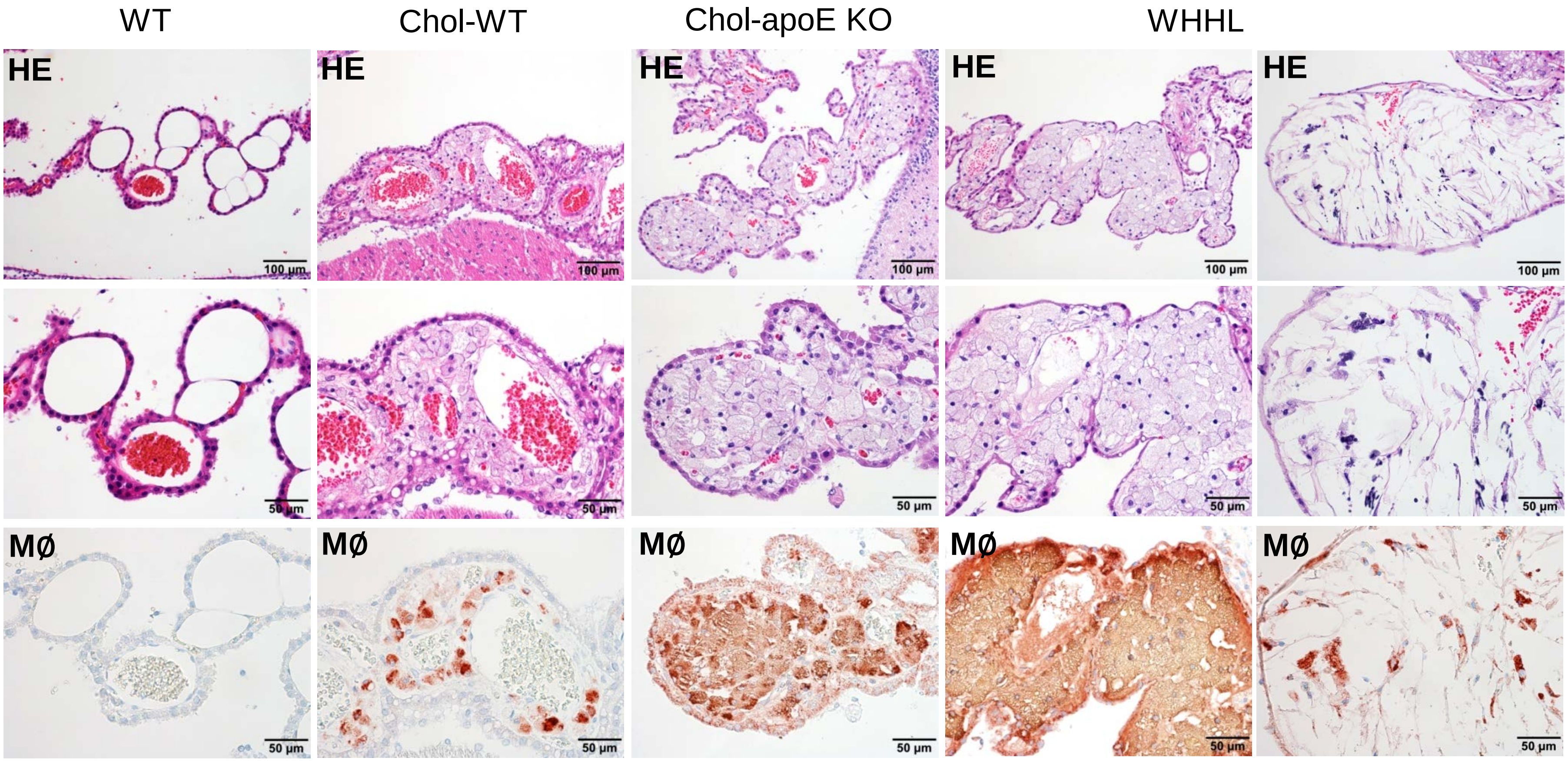

S-Figure 2. Foam cell accumulation in the choroid plexus in cholesterol-fed WT, apoE KO, and WHHL rabbits. The specimens are stained with HE or immunohistochemically stained with a monoclonal antibody against rabbit macrophages (Mϕ).

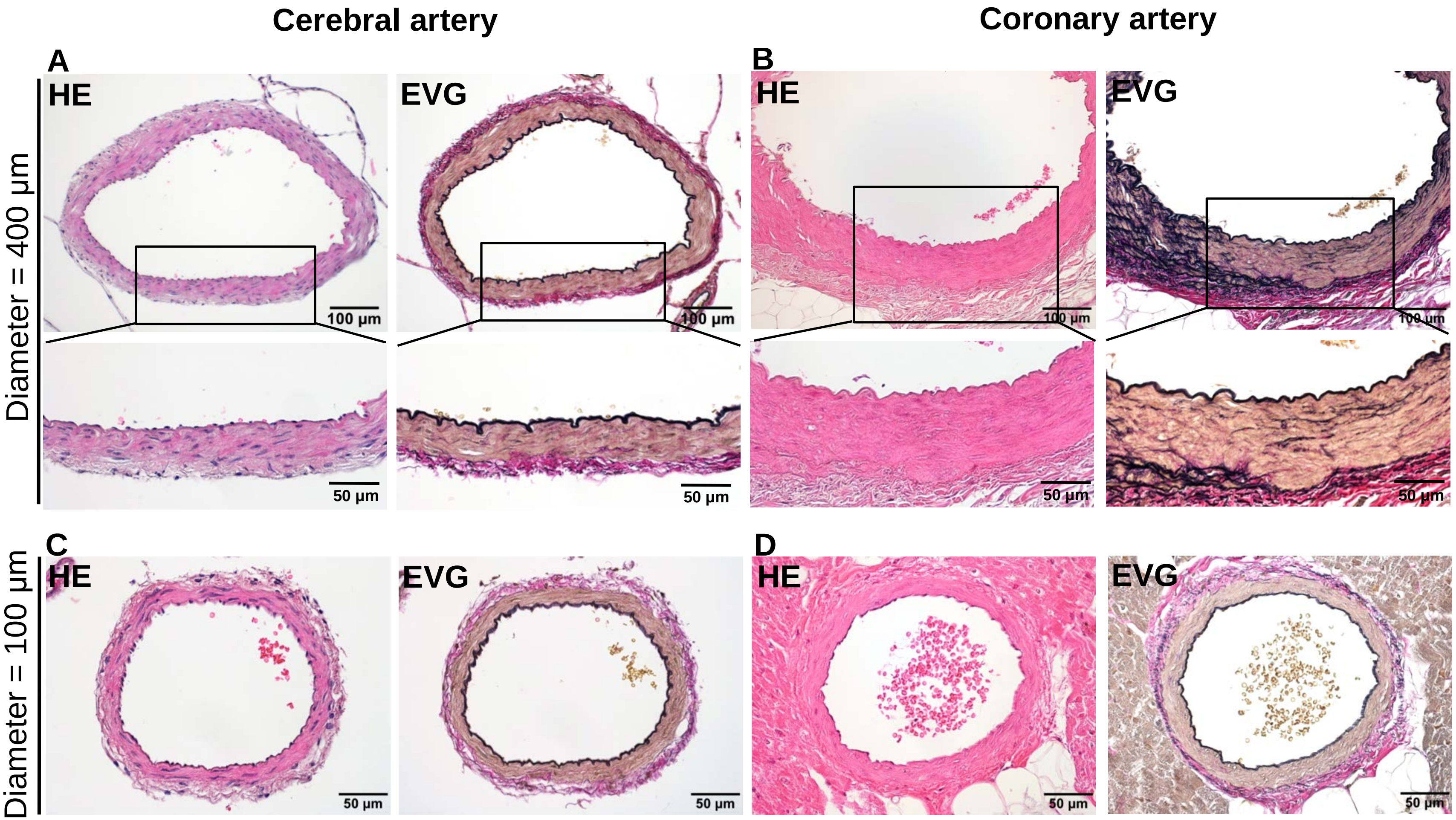

S-Figure 3. Histological features of rabbit cerebral arteries and coronary arteries. The sections are stained with HE and EVG.
